# Participatory Disease Surveillance: Engaging Communities Directly in Reporting, Monitoring, and Responding to Health Threats

**DOI:** 10.2196/publichealth.7540

**Published:** 2017-10-11

**Authors:** Mark S Smolinski, Adam W Crawley, Jennifer M Olsen, Tanvi Jayaraman, Marlo Libel

**Affiliations:** ^1^ Skoll Global Threats Fund Ending Pandemics San Francisco, CA United States

**Keywords:** public health surveillance, global health, communicable diseases, epidemiologic surveillance

## Abstract

**Background:**

Since 2012, the International Workshop on Participatory Surveillance (IWOPS) has served as an informal network to share best practices, consult on analytic methods, and catalyze innovation to advance the burgeoning method of direct engagement of populations in voluntary monitoring of disease.

**Objective:**

This landscape provides an overview of participatory disease surveillance systems in the IWOPS network and orients readers to this growing field of practice.

**Methods:**

Authors reviewed participatory approaches that include human and animal health surveillance, both syndromic (self- reported symptoms) and event-based, and how these tools have been leveraged for disease modeling and forecasting. The authors also discuss benefits, challenges, and future directions for participatory disease surveillance.

**Results:**

There are at least 23 distinct participatory surveillance tools or programs represented in the IWOPS network across 18 countries. Organizations supporting these tools are diverse in nature.

**Conclusions:**

Participatory disease surveillance is a promising method to complement both traditional, facility-based surveillance and newer digital epidemiology systems.

## Introduction

Finding outbreaks faster no matter where they first appear on the planet is a continuous challenge. New approaches to detect and monitor disease threats have emerged to supplement “traditional” disease surveillance approaches such as indicator- and facility-based surveillance (eg, notifiable diseases and laboratory tests). One of these novel approaches leverages digital connectivity to engage the public in “actively” providing public health practitioners with data that can be aggregated and analyzed for a variety of purposes including monitoring disease trends, identifying risk factors, and detection of outbreaks. This active approach of direct engagement is often referred to as participatory disease surveillance. While participatory epidemiology originated within the animal health community as a way to monitor health events in rural areas where surveillance resources are often limited, one of the first uses of crowdsourcing for public health surveillance was initiated in 2003 in the Netherlands by Science in Action [1-5].

Participatory disease surveillance collects data for public health action by directly involving the population at risk in submitting relevant data through a variety of survey tools. This can happen in many forms, from sophisticated mobile phone apps to simple hotlines. Participatory disease surveillance is “active” in the sense that it requires those who engage with the system to willingly and knowingly provide information necessary for public health action. Enlisting the help of individuals to provide data creates the potential to increase our collective understanding of disease risk and transmission patterns. Direct engagement is also an opportunity to provide information to participants about endemic disease risks and potentially enable a more rapid response to public health emergencies.

Participatory disease surveillance is considered by many to be a form of “citizen science,” though the connection to this specific term has rarely been made in the public health literature. Kullenberg et al (2016) note two general understandings of the term citizen science: one focuses on the use of public participation to collect and share data with scientists, whereas the other emphasizes a set of approaches that empower citizens to address needs or concerns in their communities [6]. These understandings are not mutually exclusive, and participatory disease surveillance as currently practiced often encompasses both.

Many participatory disease surveillance systems are structured around the reporting of syndromic information, that is, self-reported symptoms of illness rather than reports of suspected cases of a particular disease. This approach allows for expanded monitoring of the community at large, which can lead to the identification of signals of disease when coverage is sufficient. Thus, participatory disease surveillance provides a high degree of sensitivity while admittedly lacking the specificity of a laboratory test for pathogen confirmation. However, in low- and middle-income countries in particular, where traditional disease surveillance systems (including laboratory capacity) may be limited by financial and human resources, participatory disease surveillance approaches can serve as a low-cost method for routine monitoring with a sufficient level of specificity. Through this approach, unusual health events may be revealed as clusters of symptoms in both time and place. Early signals can be further investigated to verify potential health threats and compared with other surveillance systems as part of an ecosystem of public health surveillance tools.

Participatory disease surveillance can also be useful in monitoring events beyond human health symptoms. Using event-based surveillance approaches, systems have been developed, for example, that track disease vectors, report environmental hazards (or risk factors), and identify animal sickness and death in both livestock and wildlife populations [7-10].

While participatory disease surveillance methods leverage digital connectivity to directly engage the public, this is not the only approach made possible by the digital revolution. Using “big data” and computer algorithms, digital disease detection approaches seek to uncover signals of potential outbreaks and disease trends by scouring Web-based media reports, examining aggregated search queries, or analyzing social media posts [11-16]. Digital disease detection is often passive by design and may function without the direct knowledge of the user, a key difference from active, participatory disease surveillance. As such, digital disease detection has its own set of benefits and limitations. What they have in common is the “gradual” acceptance of these innovative approaches among public health authorities.

## Methods

### The International Workshop on Participatory Surveillance (IWOPS) Network

A loose collaboration of participatory disease surveillance system creators and stewards have convened periodically as the International Workshop on Participatory Surveillance (IWOPS), which met for the first time in 2012 in San Francisco, again in 2013 in Amsterdam and most recently in 2016 in Newcastle, Australia. While the IWOPS community is not an exhaustive list of relevant actors in community engagement for public health surveillance, these convenings have provided a mechanism to share best practices and insights into the evolving approach of participatory disease surveillance. This manuscript is intended to serve as an introduction to the systems and organizations that have engaged within the IWOPS community, with more detailed descriptions and results being shared throughout the accompanying articles in the theme issue of JMIR Public Health and Surveillance. The authors would like to emphasize that, even among the IWOPS community of systems, the tools and approaches described here are not exhaustive and may not fully capture the developments in this rapidly advancing practice.

Most of the IWOPS systems rely on users who volunteer to participate under the conditions of confidentiality; the systems then aggregate and map anonymous user data in an openly shared Web-based or mobile platform. Some IWOPS systems rely on trained volunteers to collect information about their communities. Table 1 outlines some of the major features of select participatory surveillance systems from the IWOPS network.

**Figure 1:**
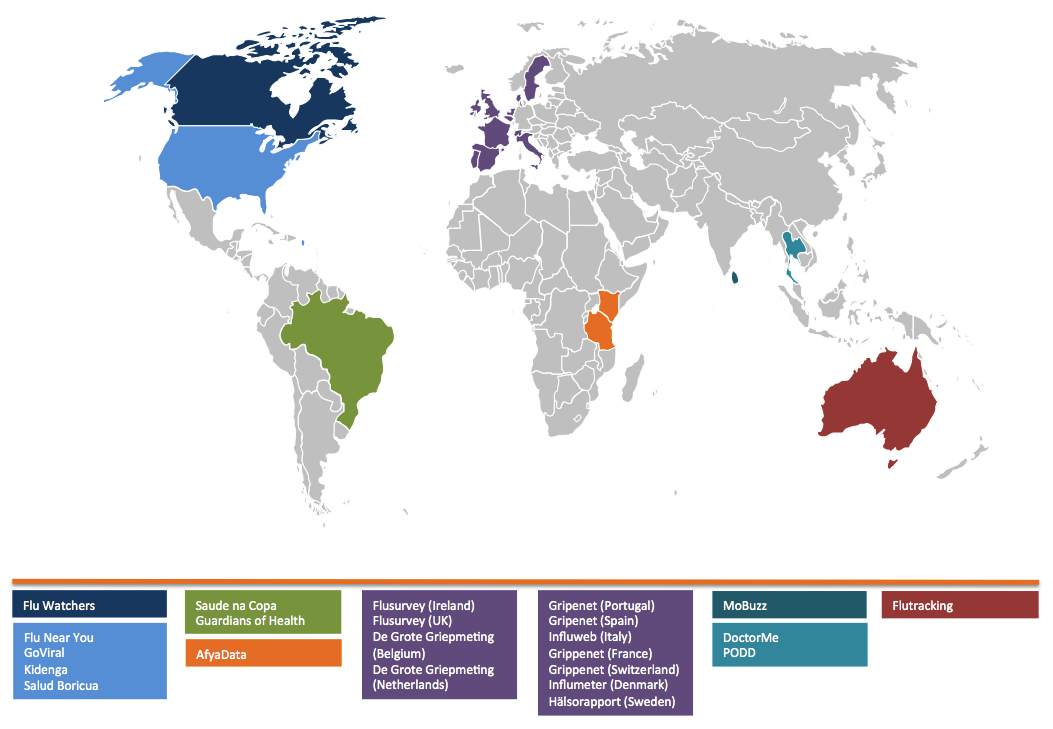
Mapping the International Workshop on Participatory Surveillance (IWOPS) participatory surveillance systems.

Participatory disease surveillance systems that monitor influenza-like illness (ILI) are prevalent in the IWOPS community, with Europe, Australia, and the United States having established such systems for many years. Several other systems have been designed with a broad list of symptoms intended to capture a range of emerging infectious diseases in humans [17-21]. Still others take an event-based approach to reporting health threats at the community level, such as the sale of counterfeit or fraudulent medications, food safety incidents, and environmental hazards like poor air and water quality [10,22]. For systems that monitor animal health events, reports may be structured to capture either illness or death of domestic or wild animals and human-animal interactions such as dog bites. Finally, some systems in the IWOPS network involve identification of potential mosquito breeding sites or other vector reporting tools to inform community control measures [7,8]. Figure 1 maps the participatory surveillance systems discussed here by country.

**Table 1:** Select participatory disease surveillance systems from the International Workshop on Participatory Surveillance (IWOPS) network.

Participatory surveillance system	System launch date	Health sector	Disease focus	Registered users	Frequency of reporting	Modality
De Grote Griepmeting (Netherlands)	November 2003	Human	ILI^a^, FBI^b^	15,000-50,000	Weekly	Website, mobile app, email
De Grote Griepmeting (Belgium)	November 2003	Human	ILI, FBI	15,000-50,000	Weekly	Website, mobile app, email
Gripenet (Portugal)	October 2005	Human	ILI	500-5000	Weekly	Website
Flutracking (Australia)	June 2006	Human	ILI	15,000-50,000	Weekly	Website, email
Influweb (Italy)	November 2008	Human	ILI, FBI	500-5000	Weekly	Website, mobile app, email
Flusurvey (England)	July 2009	Human	ILI	5000-15,000	Weekly	Website, email
Hälsorapport (Sweden)	November 2011	Human	ILI	500-5000	Monthly	Website
Flu Near You (United States)	November 2011	Human	ILI	>50,000	Weekly	Website, mobile app
Grippenet (France)	December 2011	Human	ILI	5000-15,000	Weekly	Website, email
Gripenet (Spain)	October 2012	Human	ILI	500-5000	Weekly	Website, mobile app
Salud Boricua (Puerto Rico)	October 2012	Human	ILI, VBD^c^	500-5000	Weekly	Website
Influmeter (Denmark)	October 2013	Human	ILI	500-5000	Weekly	Website, email
Flusurvey (Ireland)	November 2013	Human	ILI	500-5000	Weekly	Website, email
Saúde na Copa (Brazil)	May 2014	Human	ILI, VBD, FBI	500-5000	Daily	Website, mobile app
Doctorme (Thailand)	July 2014	Human	ILI	15,000-50,000	Daily	Mobile app
Participatory One Health Disease Detection (Thailand)	January 2015	Human or animal or environment	All syndromes; livestock outbreaks; natural disasters	500-5000	Event-based, periodic	Mobile app
FluWatchers (Canada)	November 2015	Human	ILI	<500	Weekly	Website, email
Guardiões da Saúde (Brazil)	March 2016	Human	ILI, VBD, FBI	500-5000	Daily	Website, mobile app
Mo-Buzz (Sri Lanka)	March 2016	Human or environment	VBD, breeding sites, environmental pollution	<500	Daily	Mobile app
AfyaData (Tanzania)	July 2016	Human or animal or environment	All syndromes; livestock outbreaks; wildlife outbreaks	<500	Event-based, periodic	Website, mobile app
Kidenga (United States)	August 2016	Human or environment	VBD, breeding sites	<500	Weekly	Mobile app
Grippenet (Swiss-German)	December 2016	Human	ILI	<500	Weekly	Website, email
Grippenet (Swiss-French)	December 2016	Human	ILI	<500	Weekly	Website, email

^a^ILI: influenza-like illness.

^b^FBI: foodborne illness.

^c^VBD: vector-borne disease.

## Results

### Syndromic Systems in IWOPS

Participatory disease surveillance systems began to proliferate in 2003 with the creation of “De Grote Griepmeting” in The Netherlands and Belgium by the organization Science in Action [5]. Over time this effort grew into the “Influenzanet” consortium that now includes 10 countries in Western Europe, coordinated by the ISI Foundation in Italy [18,23]. In 2006, the “FluTracking” program began in Australia and now operates as a joint initiative of Newcastle University, Hunter New England Population Health, and the Hunter Medical Research Institute [17,24]. Inspired by these systems, the Skoll Global Threats Fund (SGTF) and HealthMap of Boston Children’s Hospital partnered to launch “Flu Near You” in the United States in 2011 [19,25]. All three systems aim to track ILI through capturing symptom-based reports from volunteers on a weekly basis and have demonstrated a strong correlation with trends seen in traditional influenza surveillance systems in their respective countries [18,19,26,27]. Since Fall 2015, the Public Health Agency of Canada has been piloting a similar influenza-focused system, the “FluWatchers” platform [28]. Data being collected by these systems vary from basic demographics and symptoms of illness, such as with Flu Near You, to risk factors that may be relevant to understanding disease transmission (eg, health-seeking behavior and vaccine status), which “Influenzanet” collects through use of more robust questionnaires. “FluTracking” includes self-reported laboratory diagnosis among the data collected in their system.

In late 2012, “Salud Boricua” was developed specifically for Puerto Rico as an expansion to “Flu Near You” through a collaboration between SGTF, HealthMap, the Department of Health of Puerto Rico, and the US Centers for Disease Control and Prevention (CDC) *.* “Salud Boricua” maintained a similar design and interface to “Flu Near You,” with additional symptoms to track influenza and two other febrile illnesses: dengue and leptospirosis [29]. Another approach to broadening the scope of citizen-reported symptom data beyond an influenza focus was developed with the expansion of the “DoctorMe” mobile app in Thailand in 2014. As a preexisting health app available via Web and mobile devices, “DoctorMe” added a mechanism for volunteers to report on symptoms of disease, leveraging the popularity of the “DoctorMe” app and its utility for diagnosing potential maladies [20,30].

International mass gatherings have become a focal point for disease surveillance and pandemic prevention. The 2014 World Cup tournament that took place in Brazil provided an opportunity to test the use of participatory disease surveillance tools in the context of such gatherings. The Brazilian Ministry of Health partnered with SGTF and Epitrack to create and deploy “Saúde na Copa” (healthy cup), a smartphone app that encouraged users to report healthy status or symptoms of illness on a daily basis throughout the tournament; a first attempt at using this approach in a mass-gathering setting. Encouraged by the success of this technology, the same partners created “Guardiões da Saúde” (guardians of health) for use during the 2016 Olympic and Paralympic Games in Rio de Janeiro [21,31]. Today, “Guardiões da Saúde” continues as a complement to routine disease surveillance throughout Brazil.

Vector-borne diseases have also emerged as another use case for public reporting of health threats in the wake of the recent and rapid spread of chikungunya and Zika viruses in the Western Hemisphere, in addition to the ongoing burden of dengue worldwide. As a result, community-reporting applications such as “MoBuzz” and “Kidenga” have been deployed in Sri Lanka and the southern United States, respectively [7,8,32]. Both systems seek to not only leverage community participation in reporting and tracking of symptoms but also to provide education on prevention strategies such as personal protection and disruption of mosquito breeding environments.

### Event-Based and One Health Surveillance Systems in IWOPS

Participatory surveillance approaches are not limited only to the symptom-based reporting model. The AfyaData system in Tanzania and the Participatory One Health Disease Detection (PODD) system in Thailand both apply event-based surveillance models with a One Health focus to community reporting for human, animal, and environmental health events [9,10,22]. These systems leverage the use of trained volunteers (rather than general public crowdsourcing) in local communities to report on health events that range from suspect cases of dengue fever, to disease outbreaks in livestock, to contamination of water sources. Both systems have developed strong partnerships with regional or national government authorities to ensure that threats reported through these novel systems can be acted upon in a timely manner by relevant health authorities.

### Integration of Modeling and Forecasting in IWOPS

As the many surveillance systems in the IWOPS community continue to improve our understanding of disease transmission and spread, opportunities are increasing to leverage these datasets for the modeling and forecasting of disease trends and for anticipating health threats before they emerge. The MoBuzz system integrates predictive analytics to provide feedback on vector hotspots to users [32]. HealthMap’s “FluTrends” tool integrates data from “Flu Near You” alongside other datasets, and “Influenzanet” data feeds into the “FluOutlook” platform; both efforts seek to model the spread of influenza and provide forecasting of peak ILI activity [33-36].

## Discussion

### Benefits of Participatory Disease Surveillance

Foremost among the benefits of participatory surveillance is the ability to conduct large scale, population-based monitoring at low cost. As the number of users in a system increases, the sensitivity afforded by the expanded monitoring of participatory surveillance increases as well. Additionally, in some contexts, traditional health care-based surveillance methods may underestimate the true disease burden in the population due to a dependence on health care-seeking behavior on the part of the individual. In contrast, participatory surveillance systems can engage people who may not interact with a health care provider due to lack of access, resource constraints, or cultural norms. If participation is high enough and reflective of the larger population, participatory surveillance provides an opportunity to develop a more complete estimate of disease burden in a population in complement with sentinel provider networks [37]. Thus, capturing data on the general population, many of whom may not be represented in other surveillance systems, can bring significant benefit, especially when performed with the speed enable by digital reporting.

Participatory disease surveillance may also provide insights about user health behaviors. For example, systems such as “Influenzanet” are able to identify certain behavioral risk factors for ILI, assess attitudes toward influenza vaccination among pregnant women, estimate health care-seeking behavior during a pandemic, and examine social contact patterns [38-41]. Both “FluTracking” and “Influenzanet” researchers have also endeavored to leverage their platforms to provide measures of field vaccine effectiveness [27,42-45]. The advantage of this approach is found in rapidly capturing vaccine data on the general population, many of whom may not otherwise be evaluated, while the lack of laboratory or case confirmation limits the inferences that can be made from this information.

As participatory systems grow over time, the need to identify characteristics of participants that contribute consistently has become a priority. Fortunately, these characteristics can be continually assessed and approaches revised to ensure strong participation from across all segments of a population. In one study of “Influenzanet” participants, it was determined that lower participation was associated with characteristics such as lower educational status, smoking, younger age, not being vaccinated against seasonal influenza, and living in a household with children [40]. A study of “Flu Near You” participants found similar results for the effect of age but noted that users reporting on behalf of household members (who are often children) were more likely to be consistent reporters than other participants [46]. These findings might allow public health staff to increase promotion efforts to populations with low levels of participation in order to achieve a reporting population that reflects the general public. Understanding motivations for participation is also critical. Results from a survey targeting Dutch “Influenzanet” participants, for example, showed that the desire to contribute to a scientific goal is the most important motivator for all types of participants and that availability of scientific information and data are important for learning [47]. Although it is likely that factors affecting participation for different systems vary by nation and culture, these insights provide grounds for hypothesis testing and refining recruitment and retention practices.

The potential for rapid two-way communication between health authorities and participatory disease surveillance system users provides another important opportunity for public health messaging and education. As users are actively engaged in providing information to the system, opportunities exist to inform users about disease activity levels in their neighborhoods, provide automatic messaging back to volunteers and local authorities, and share appropriate prevention and control measures during disease outbreaks or other health emergencies. Having a way for health authorities to message a large population of volunteer users, which may include hard-to-reach populations, can be especially valuable for disease control and prevention activities. Many participatory disease surveillance systems have included useful information for the user, such as the location of vaccine distributors and mapping of disease activity [19,29]. Others have included health quizzes and other gamification approaches to increase user engagement and improve health promotion, while targeted alerts are used in some systems to trigger local government health interventions for the reporting population [9,10,20,31,32]. Though the degree to which IWOPS systems provide feedback to users varies greatly, it is likely that this mechanism will continue to be leveraged to provide greater value to users and increase participation in these systems.

Finally, participatory disease surveillance provides flexible data systems and user interfaces that enable health authorities to rapidly modify the data elements being collected and disseminate information in near-real time. For syndromic systems, new symptoms can be added if an emerging infectious disease is associated with additional symptoms not currently collected. “Flu Near You,” for example, tested the addition of new symptoms related to dengue and Zika viruses as these diseases became more prevalent in the United States. For event-based systems, the addition of new types of health threats that may emerge allows health agencies to be more responsive to community needs or concerns.

### Challenges of Participatory Disease Surveillance

Perhaps the most consistent challenge for participatory disease surveillance systems is the recruitment and retention of participants of a demographic mix that reflects the population at risk. Marketing and recruitment efforts have had varying levels of success. “FluTracking,” which has been very successful in growing and maintaining a large volunteer network, has employed a number of tactics where they have found success; these include using friend-referral emails, inviting users to report for household members, and refraining from the use of barriers such as usernames and passwords [48]. “Mo-Buzz” incentivizes reports that were submitted by public health inspectors (PHIs), with the total number of reports submitted by an investigator contributing toward their yearly performance bonuses and pay increments [49]. “Flu Near You” has experimented with paid marketing efforts and social media campaigns with some success. The cost of some marketing tactics such as online ad buys, however, may adversely impact the low-cost nature of participatory disease surveillance. The efficient use of marketing efforts combined with smart design principles and a user-friendly approach may help sustain participation in these systems over time.

Symptom-based, self-reporting systems lack the ability to determine the causative agent of each reported syndrome, a limitation shared by many sentinel provider networks that may only test a limited number of patients. For systems focused on monitoring ILI, several pathogens may cause the nonspecific symptoms that comprise an ILI definition. With this in mind, the “GoViral” study was launched in late 2013 to compare acquired community generated diagnostic samples with participatory symptom reporting. Users are enrolled into a self-reporting system, sent a home testing kit, and instructed to perform specimen collection and a rapid diagnostic test within 48 hours of experiencing any flu symptoms. The study, which has expanded beyond its original target sites in Massachusetts and has collected hundreds of samples to date, may serve as a model for improving linkages between participatory systems and laboratory diagnostics [50,51]. Those linkages will allow participatory disease surveillance to increase in specificity as home test kits and rapid diagnostics increase in availability and accuracy and decrease in cost.

Not every system within the IWOPS network is established enough to draw firm conclusions on user characteristics, especially as the growth trajectory of users in many systems is on a steady increase. The behaviors and characteristics identified through “Flu Near You” and “Influenzanet,” for example, indicate that participants tend toward higher socioeconomic status and healthier behaviors than the general populations in their respective countries [40,46]. Whether these trends will continue to hold true as recruitment efforts increase and whether similar trends in other regions of the world are present, remain to be explored. Other questions include whether reporting rates increase when an outcome of interest is present for volunteers (eg, experiencing symptoms and finding a dead chicken) as opposed to when users would otherwise submit a “zero report” to confirm no event or the absence of any symptoms.

While participatory disease surveillance has been increasingly accepted by public health agencies, a continued effort to integrate these data sources into broader disease surveillance frameworks and public health decision-making processes remains a challenge. Identifying how these systems contribute to effective public health action in various contexts will be an ongoing effort. Certain event-based tools can provide concrete case studies—such as when PODD was used to detect and control a backyard chicken outbreak in Chiang Mai, Thailand—and health agencies’ use of syndromic tools like “Influenzanet” and “FluTracking” for monitoring and situational awareness can be documented as successful examples [22]. Health agencies should approach the adoption of participatory disease surveillance tools with the aim of integrating insights from multiple, complementary data sources, recognizing that each have their own underlying populations and data collection methods that contain specific biases. As noted by Leal-Neto et al (2017), collaborations in this space often need government engagement to be successful. It is vital that the role of government health agencies and partner organizations such as telecommunication companies, is clear from the outset [31].

Evaluative methods for judging the quality, timeliness, or representativeness of information returned by participatory systems must continue to aid the evolution, adoption, and integration of these systems as part of routine health monitoring in the community. This is made difficult by a range of variables such as population size, reporting consistency, and balancing the amount of data gathered on users while still respecting privacy. Practitioners and evaluators must continue to find ways to improve evaluative approaches and identify outcomes that are indicative of success. Potential measurable outcomes include the volume of reports submitted, the system’s role in initiating or accelerating responses, and the system’s influence on behaviors and population health outcomes [52]. The role or roles that participatory disease surveillance can play in overall community health monitoring need to be more clearly defined to properly evaluate performance against stated aims.

### Future Opportunities

Innovative surveillance approaches are increasingly needed to provide public health officials, from the local to global, with scalable, affordable, and flexible tools that enable population-based disease monitoring for prevention and control of emerging health threats. Both the International Health Regulations (IHR) and the Global Health Security Agenda (GHSA) have mandated that countries develop the ability to rapidly report emerging events [53]. The GHSA “Real-Time Surveillance” Action Packages Detect 2 and 3 specifically focus on “real-time surveillance.” These action packages call for interoperable, electronic systems with the capability to detect health threats through both syndromic and event-based surveillance [54]. These aims could be well served by the inclusion of participatory disease surveillance methods in countries seeking to meet these targets.

To realize their full potential, participatory tools must be extended to all communities, not simply those with reliable Internet connections and high rates of smartphone penetration. It is no secret that some of the most at-risk communities are those furthest removed from the reach of health authorities. Efforts must be made to connect rural and low-income populations to health systems and surveillance networks. In doing so, opportunities exist to improve populations’ health literacy in terms of both understanding disease risk in their community and receiving feedback about preventive actions.

The promise of participatory disease surveillance may only be fully realized when it becomes an integrated component of a surveillance ecosystem that includes data from health facilities, sentinel surveillance systems, digital disease detection tools, and other sensors such as wearable technology and wireless thermometers. Additionally, as integration of disparate data sources becomes more viable, exploration into the value of linking self-reported data with electronic medical records may also yield significant returns. As participatory disease surveillance continues to emerge as a community of practice, continued knowledge sharing around best practices and lessons learned should be sustained. The authors hope that the IWOPS community described in this manuscript and throughout the theme issue of JMIR Public Health and Surveillance can serve as one such vehicle as we all endeavor to improve disease surveillance.

## Abbreviations

CDC: Centers for Disease Control and Prevention
GHSA: Global Health Security Agenda
IHR: International Health Regulations
ILI: influenza-like illness
IWOPS: International Workshop on Participatory Surveillance
PHI: public health inspector
PODD: Participatory One Health Disease Detection
SGTF: Skoll Global Threats Fund
